# Utilizing convolutional neural networks to classify monkeypox skin lesions

**DOI:** 10.1038/s41598-023-41545-z

**Published:** 2023-09-03

**Authors:** Entesar Hamed I. Eliwa, Amr Mohamed El Koshiry, Tarek Abd El-Hafeez, Heba Mamdouh Farghaly

**Affiliations:** 1 Department of Mathematics and Statistics, College of Science, King Faisal University, P.O. Box: 400, 31982 Al-Ahsa, Saudi Arabia; 2 Department of Computer Science, Faculty of Science, Minia University, Minya, Egypt; 3 Present Address: Department of Curricula and Teaching Methods, College of Education, King Faisal University, P.O. Box: 400, 31982 Al-Ahsa, Saudi Arabia; 4 Faculty of Specific Education, Minia University, Minya, Egypt; 5 Computer Science Unit, Deraya University, New Minya, Egypt

**Keywords:** Bioinformatics, Computer science

## Abstract

Monkeypox is a rare viral disease that can cause severe illness in humans, presenting with skin lesions and rashes. However, accurately diagnosing monkeypox based on visual inspection of the lesions can be challenging and time-consuming, especially in resource-limited settings where laboratory tests may not be available. In recent years, deep learning methods, particularly Convolutional Neural Networks (CNNs), have shown great potential in image recognition and classification tasks. To this end, this study proposes an approach using CNNs to classify monkeypox skin lesions. Additionally, the study optimized the CNN model using the Grey Wolf Optimizer (GWO) algorithm, resulting in a significant improvement in accuracy, precision, recall, F1-score, and AUC compared to the non-optimized model. The GWO optimization strategy can enhance the performance of CNN models on similar tasks. The optimized model achieved an impressive accuracy of 95.3%, indicating that the GWO optimizer has improved the model's ability to discriminate between positive and negative classes. The proposed approach has several potential benefits for improving the accuracy and efficiency of monkeypox diagnosis and surveillance. It could enable faster and more accurate diagnosis of monkeypox skin lesions, leading to earlier detection and better patient outcomes. Furthermore, the approach could have crucial public health implications for controlling and preventing monkeypox outbreaks. Overall, this study offers a novel and highly effective approach for diagnosing monkeypox, which could have significant real-world applications.

## Introduction

Monkeypox is an uncommon viral disease caused by the monkeypox virus (MPXV). Its first identification was in monkeys in the 1950s in the Democratic Republic of Congo, and later in humans in 1970. The disease is endemic in Central and West Africa, with sporadic outbreaks reported in other regions globally, including the United States, Europe, and Asia^1,2^. The clinical symptoms of monkeypox are similar to those of smallpox and can include fever, rash, and pustules. However, monkeypox is generally less severe than smallpox, with a lower mortality rate^3^.

The diagnosis of monkeypox is usually made based on clinical presentation and laboratory tests. One of the key laboratory tests used for diagnosis is the detection of the virus in skin lesions using polymerase chain reaction (PCR) or other methods. However, the interpretation of these tests can be challenging, as other viruses, such as varicella-zoster virus and herpes simplex virus, can cause similar lesions^4,5^.

Artificial intelligence (AI) techniques, such as machine learning and deep learning, have garnered significant attention in recent years for medical image analysis. These techniques have demonstrated promise in various applications, including the diagnosis of skin diseases. Among them, Convolutional Neural Networks (CNNs) have been particularly successful in image analysis tasks, providing a powerful tool for medical image analysis^6^.

The utilization of CNNs in classifying skin lesions poses several challenges^7^. First, there is limited availability of large and high-quality datasets of monkeypox skin lesions, which makes it difficult to train and validate the performance of the CNN models. Second, monkeypox lesions may vary in size, shape, color, texture, and location on the body, which can affect the accuracy of the CNN models in correctly identifying the lesions^8,9^. Third, some lesions may have overlapping features with other skin conditions or diseases, which can result in misclassification or confusion by the CNN models. Fourth, the CNN models may require significant computational resources and expertise to train and optimize, which can limit their accessibility and usability for researchers and clinicians with limited resources or expertise in machine learning. Lastly, the CNN models may require further validation and testing on larger and more diverse datasets to ensure their reliability, generalizability, and robustness in real-world clinical settings^10^.

### Aim

The primary aim of this study is to devise a precise and dependable algorithm for the automated classification of monkeypox skin lesions using CNNs and GWO optimization. The successful classification of monkeypox skin lesions can aid in the early detection, diagnosis, and treatment of the disease, ultimately resulting in improved patient outcomes.

### Objectives

The objectives of this study are to propose a novel approach for classifying monkeypox skin lesions using CNNs and to develop a reliable and accurate model for this purpose. The study aims to reduce the burden of manual diagnosis of monkeypox skin lesions, which can be time-consuming and prone to errors. It also seeks to provide a cost-effective and accessible alternative to traditional diagnostic methods that often require specialized training and equipment. By enabling faster and more accurate diagnosis, the proposed approach could improve the early detection and treatment of monkeypox. The study compares the performance of the CNN model with and without the GWO optimizer for monkeypox classification and demonstrates the effectiveness of GWO optimization in improving the performance of CNN models for similar classification tasks. The proposed approach using CNNs and GWO optimization significantly improves the accuracy of monkeypox skin lesion classification. It has potential benefits for improving the accuracy and efficiency of monkeypox diagnosis and surveillance, enabling faster and more accurate diagnosis of monkeypox skin lesions, potentially leading to earlier detection and better patient outcomes. The study's findings could have crucial public health implications for controlling and preventing monkeypox outbreaks.

The main contribution of this paper can be summarized as follows:

- Proposal of a novel CNN-based approach for classifying monkeypox skin lesions.
- Development of an accurate CNN model for monkeypox skin lesion classification.
- Reduction of the burden of manual monkeypox diagnosis which can be time-consuming and error-prone.
- Provision of a cost-effective and accessible alternative to traditional monkeypox diagnostic methods.
- Improvement of early monkeypox detection and treatment by enabling faster and more accurate diagnosis.
- Evaluation of the performance of the approach on a test set using metrics such as accuracy, precision, recall, and F1-score.
- Comparison of the performance of the CNN model with and without the GWO optimizer for monkeypox classification.
- Demonstration of the effectiveness of GWO optimization for improving CNN models for such classification tasks.
- Finding that the proposed CNN and GWO approach can significantly improve monkeypox skin lesion classification accuracy.
- Highlighting the potential benefits of the proposed approach for improving monkeypox diagnosis and surveillance accuracy and efficiency.
- Enabling faster and more accurate diagnosis of monkeypox skin lesions, potentially leading to earlier detection and better patient outcomes.
- Crucial public health implications for controlling and preventing monkeypox outbreaks.

The organization of the paper includes the related work in “Related work”. The preliminaries and methodologies of the proposed monkeypox skin lesions based on the CNN approach is in “Preliminaries” and “Methodology”. The experimental results and discussion are investigated in “Experimental results and analysis” and “Discussion”. The future direction and conclusions are demonstrated in “Future direction” and “Conclusion”.

## Related work

The world has been hit hard by a multinational monkeypox outbreak, which has come at a time when the world is still reeling from the COVID-19 pandemic. By the end of June 2022, there was a sudden and significant increase in the number of non-endemic human monkeypox cases, with over 4900 instances reported across the Western Hemisphere. The virus has spread from its initial exposure in Africa to human-to-human transmission within each affected country. Virologists have identified two distinct variants of the monkeypox virus—the Central Africa clade and the West Africa clade. As of now, there is no proper treatment available for the monkeypox virus^11,12^. While the mortality rates for monkeypox are generally low^13^, early detection is crucial for implementing effective containment measures such as patient isolation and contact tracing to prevent its spread. Clinical identification of monkeypox can be challenging due to its similarity with other pox viruses. Diagnosis typically involves examining skin lesions and evaluating the patient's exposure history, followed by testing the lesions using dermatoscopic images and confirming the diagnosis using polymerase chain reaction (PCR) testing^14^.

Physicians can improve their accuracy in diagnosing skin cancer, skin lesions, and psoriasis with the help of classification models^9^. Deep CNNs have proven to be effective in performing general and highly variable tasks across various categories^15,16^. Researchers have trained CNNs using large datasets of skin lesion images for binary and multiclass classification, achieving performance comparable to or superior to that of board-certified specialists and dermatologists^17^

To select optimal parameters for a model, researchers typically perform mathematical modeling and optimization using an optimization method. Metaheuristic algorithms, including various ensemble techniques, have been extensively employed for solving classification problems due to their ability to deal with complex, multi-dimensional, and ill-behaved optimization problems and provide satisfying results in a reasonable time^18–20^.

Various AI models have been proposed for different applications, and combining the attributes of different models can create an ensemble prediction model using ensemble techniques^21, 22^. Resampling the training set is one of the more efficient methods, while others employ different prediction algorithms and adjust predictive strategy parameters. To aggregate the predictions, an ensemble of techniques is utilized^23,24^.

The Al-Biruni Earth radius (BER) optimization technique has been proposed as a new optimization algorithm for solving classification problems^25,26^. It's successful balancing of exploration and exploitation is a significant advantage, but it performs worse when more variables are used. To overcome this limitation, the BERSFS algorithm combines the benefits of the BER algorithm with the stochastic fractal search (SFS) algorithm^27^, which has a simple usage but can experience performance issues with a large number of local optimum solutions.

Doaa Sami et al.^14^ proposed the use of AI methods to diagnose monkeypox using a digital skin image classification algorithm. They suggested that artificial neural networks (ANNs) could be particularly effective in detecting monkeypox by analyzing and processing skin images. This is because ANNs can learn important features from complex data during the training stage, making them ideal for diagnosing skin lesions.

Veysel Harun Sahin et al.^28^ have developed a mobile system that can automatically detect human monkeypox skin lesions. To achieve this goal, they first trained a deep transfer learning-based system using images from the MSLD database. In this stage, they retrained various pre-existing networks using the transfer learning approach and compared their results. After evaluation, they selected MobileNetv2, which achieved an accuracy of 91.11%, as the best-performing network and adapted it into an Android mobile application. The proposed system was then compared with other studies that used the same database and was found to produce better results.

Diponkor Bala et al.^29^ developed an advanced deep learning-based method using a first-ever database called "MSID" to detect and classify monkeypox disease early on. They applied an augmentation technique to increase the number of images in the dataset and presented a modified DenseNet-201-based deep CNN model called "MonkeyNet" for multiclass classification of monkeypox from skin images. The model achieved high accuracy, with 93.19% and 98.91% in the multiclass classification of the original and augmented datasets, respectively. The proposed model could be implemented in a reliable mobile application to support medical personnel in diagnosing monkeypox disease. The study has the potential to improve knowledge and diagnosis of monkeypox disease, and future work could involve expanding the study to include a larger number of clinical data and skin images.

Table 1 shows the detailed results obtained by various Convolutional Neural Networks (CNN) models for a specific classification task. The models were compared based on their accuracy, sensitivity, specificity, F1 score, training time, and size of model weight file.

**Table 1:** Results obtained by CNN models^30^.

Models	Accuracy	Sensitivity	Specificity	F1 Score	Training time	Size of model weight file
ResNet-18	98.25%	96.55%	100.00%	98.25%	3 min 32 s	42.7 Megabyte
ResNet-50	96.49%	93.10%	100.00%	96.43%	4 min 33 s	90.0 Megabyte
VGG-16	92.98%	89.66%	96.43%	92.86%	5 min 39 s	512 Megabyte
Densenet-161	96.49%	96.55%	96.43%	96.55%	6 min 52 s	102 Megabyte
EfficientNet B7	94.74%	100.00%	89.29%	95.08%	8 min 27 s	245 Megabyte
EfficientNet V2	96.49%	100.00%	92.86%	96.67%	8 min 57 s	449 Megabyte
GoogLeNet	96.49%	96.55%	96.43%	96.55%	5 min 35 s	512 Megabyte
MobileNet V2	98.25%	96.55%	100.00%	98.25%	3 min 42 s	8.75 Megabyte
MobileNet V3	75.44%	62.07%	89.29%	72.00%	3 min 10 s	5.94 Megabyte
ResNeXt-50	92.98%	100.00%	85.71%	93.55%	5 min 15 s	88.0 Megabyte
ShuffleNet V2	78.95%	65.52%	92.86%	76.00%	3 min 37 s	20.6 Megabyte
ConvNeXt	96.49%	100.00%	92.86%	96.67%	23 min 25 s	748 Megabyte

Table 2 provides a comparative analysis of the relevant studies of monkeypox detection using deep learning methods. The table includes the authors' names and publication year, the purpose of the study, the proposed methodology, key parameters, and the models used in each study. The scores achieved by each study are also presented and discussed in detail in the subsequent sections of the paper. The studies included in the table are carefully selected to provide a comprehensive overview of the state-of-the-art approaches for detecting monkeypox. The comparison highlights the strengths and limitations of each study, and provides insights into the effectiveness of different methods and models used for monkeypox detection. The table serves as a useful reference for researchers and practitioners interested in this area, as it provides a clear understanding of the existing approaches and the gaps in knowledge that need to be addressed.

**Table 2:** A comparative analysis of the relevant studies of monkeypox detection using deep learning methods.

Author/year	Purpose	Proposed methodology	Key parameters	Model
Ali et al., 2022^31^	Monkeypox skin lesion detection	Utilizing deep learning models for detecting monkeypox skin lesions	F1-score	VGG-16, ResNet50, and InceptionV3 models
Situla and Sahahi, 2022^32^	Monkeypox virus detection	Detection of monkeypox virus by transfer learning methods	Accuracy and F1-score	Xception, DenseNet
Ahsan et al., 2020^33^	Detecting monkeypox disease	Image data collection and implementation of a deep learning-based model in detecting monkeypox disease	AUC	They propose and evaluate a VGG16 model with D curve
Sahin et al., 2022^28^	Human monkeypox classification from skin lesion images	Human monkeypox classification from skin lesion images with deep pre-trained network	Accuracy and F1-score	GoogleNet, EfficientNetb0, NasnetMobile, ShuffleNet, MobileNetv2 models
Hossain et al., 2022^34^	Lyme disease from skin lesion images	Convolutional neural networks with transfer learning to diagnose Lyme disease from skin lesion	AUC, sensitivity, accuracy and specificity	ResNet50
Philippe et al., 2019^35^	Automated detection of erythema migrans	Automated detection of erythema migrans and other confounding skin lesions via deep learning	AUC and accuracy	Resnet50
Proposed method	Automated classification of monkeypox skin lesions	Automated classification of monkeypox skin lesions using CNNs and GWO optimization	Accuracy Precision Recall F1 Score AUC Score	CNNs and GWO

## Preliminaries

### Convolutional neural network (CNN)

**Figure 1:**
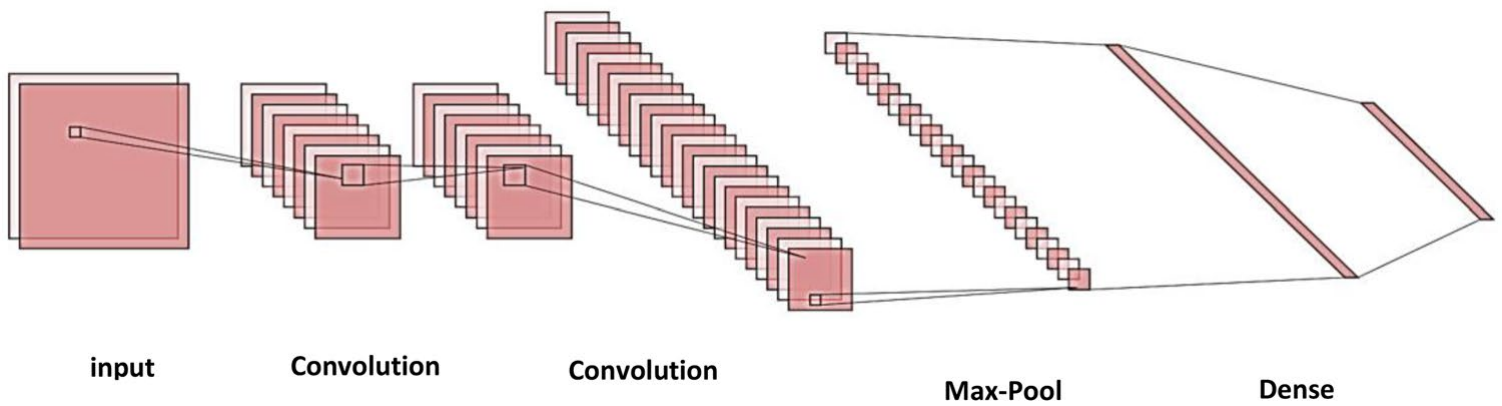
The basic CNN architecture^38^.

CNN^36,37^ is an advancement of the Multilayer Perceptron (MLP) neural network and is specifically designed to process two-dimensional data. Like any neural network, CNN has neurons with weights, biases, and activation functions. CNN can learn hierarchical representations of input data automatically, which are more robust and expressive than manually engineered features. It is composed of multiple layers of neurons, including convolutional layers, activation functions, pooling layers, and fully connected layers. In the convolutional layer, a set of filters or kernels is applied to the input data to generate feature maps that capture various aspects of the input. The activation functions introduce non-linearity to the output of each convolutional layer, while the pooling layers down sample the feature maps, reducing their size while retaining the most important features. Finally, the fully connected layers utilize the output of the previous layers to perform the final classification or regression. Figure 1 provides an essential visual representation of the building blocks of a CNN. It helps to clarify how the CNN architecture extracts features from input images and performs classification through multiple convolutional and pooling layers, as well as fully connected layers.

### Grey wolf optimization (GWO)

GWO is a nature-inspired metaheuristic algorithm used for solving optimization problems^39^. It can be applied to solve optimized problems and achieves excellent results^40,41^. It is based on the social hierarchy and hunting behavior of gray wolves in the wild. To simulate the leadership hierarchy, there are four types of grey wolves which are alpha (α), beta (β), delta (δ), and omega (ω). Those four types can be used for simulating the leadership hierarchy. The hunting (optimization) is guided by three wolves (α, β, and δ). The ω wolves follow them^42^. During the hunting process, it is known that grey wolves surround their prey. Mathematically, this is modeled by Eqs. (1) and (2) ^40, 41^:

1 $$\overrightarrow{F}= \left|\overrightarrow{K}.{\overrightarrow{Y}}_{n}\left(s\right)-\overrightarrow{Y}\left(s\right)\right|,$$

2 $$\overrightarrow{Y}\left(s+1\right)= \left|\overrightarrow{K}.{\overrightarrow{Y}}_{n}\left(s\right)- \overrightarrow{B}.\overrightarrow{F}\right|,$$

where s denotes the current iteration, $\overrightarrow{B}$ and $\overrightarrow{K}$ are coefficient vectors, $\overrightarrow{Y}$ n is the vector of the prey position, and represents the vector of the grey wolf position. Equations (3) and (4) can be used to calculate the coefficient vectors $\overrightarrow{B}$ and $\overrightarrow{K}$, respectively.

3 $$\overrightarrow{B}= 2.\overrightarrow{ b}.{\overrightarrow{l}}_{1}-\overrightarrow{b},$$

4 $$\overrightarrow{K}= 2.{\overrightarrow{l}}_{2},$$

where components of b are gradually reduced from 2 to 0 during the iterations, while l_1_, and l_2_ are vectors with random values within the range of 0 to 1.

To simulate the hunting process of grey wolves, it is assumed that α (the most promising candidate solution), δ, and β have greater knowledge about the possible location of prey. Therefore, the three best solutions obtained so far are saved, and other search agents (including ω) are required to adjust their positions based on the positions of the best search agents. Equations (5), (6), and (7) are utilized to update the positions of the grey wolves^4, 5^:

5 $$\overrightarrow{{F}_{\alpha }}= \left|\overrightarrow{{K}_{1}}.{\overrightarrow{Y}}_{\alpha }-\overrightarrow{Y}\right|, \overrightarrow{{F}_{\beta }}= \left|\overrightarrow{{K}_{2}}.{\overrightarrow{Y}}_{\beta }-\overrightarrow{Y}\right|, \overrightarrow{{F}_{\delta }}= \left|\overrightarrow{{K}_{3}}.{\overrightarrow{Y}}_{\delta }-\overrightarrow{Y}\right|,$$

6 $$\overrightarrow{{Y}_{1}}= \overrightarrow{{Y}_{\alpha }}-\overrightarrow{ {B}_{1}}.({\overrightarrow{F}}_{\alpha }), \overrightarrow{{Y}_{2}}= \overrightarrow{{Y}_{\beta }}-\overrightarrow{ {B}_{2}}.({\overrightarrow{F}}_{\beta }), \overrightarrow{{Y}_{3}}= \overrightarrow{{Y}_{\delta }}-\overrightarrow{ {B}_{3}}.({\overrightarrow{F}}_{\delta }),$$

7 $$\overrightarrow{Y}\left(s+1\right)=\frac{\overrightarrow{{Y}_{1}}+\overrightarrow{{Y}_{2}}+\overrightarrow{{Y}_{3}}}{3}.$$

### Motivation and problem formulation

In this research, identifying the hyper-parameters of CNN is defined as an optimization problem; in which the parameters are represented by a list of real numbers. The objective is to optimize the hyper-parameters using GWO^43^ and then use them for monkeypox classification to achieve more classification accuracy. The objective function is defined as follows

8 $$\mathrm{Model \; Accuracy }=\mathrm{ CNN }\left(\overrightarrow{P},\overrightarrow{WI},\overrightarrow{{TR}_{j}}\right),$$

9 $${}_{{\vec{P} \in R^{n} }}^{{MAX\;Acuracy}} {\text{CNN}}\left( {\vec{P},\overrightarrow {{WI}} ,\overrightarrow {{TR_{j} }} } \right) < j_{{maximum}} .$$

The architecture of CNN is defined by Eq. (8) which takes input vectors, $\overrightarrow{P},\overrightarrow{WI},\overrightarrow{{TR}_{j}}$, where $\overrightarrow{P}$, represents the hyper-parameter vector of k dimension, $\overrightarrow{WI}$ represents the weight vector of CNN, TR_j_ is some data selected from training data. The output of this function is the accuracy of the model. The objective function, defined by Eq. (9), seeks to maximize the accuracy of CNN for the given hyper-parameters. The parameter ${j}_{maximum}$, is set by the user to control the number of iterations required by CNN for hyperparameter optimization. A larger value of  ${j}_{maximum}$ will result in longer optimization times, so it is important for the user to set this value carefully to balance optimization time and cost-effectiveness.

## Methodology

**Figure 2:**
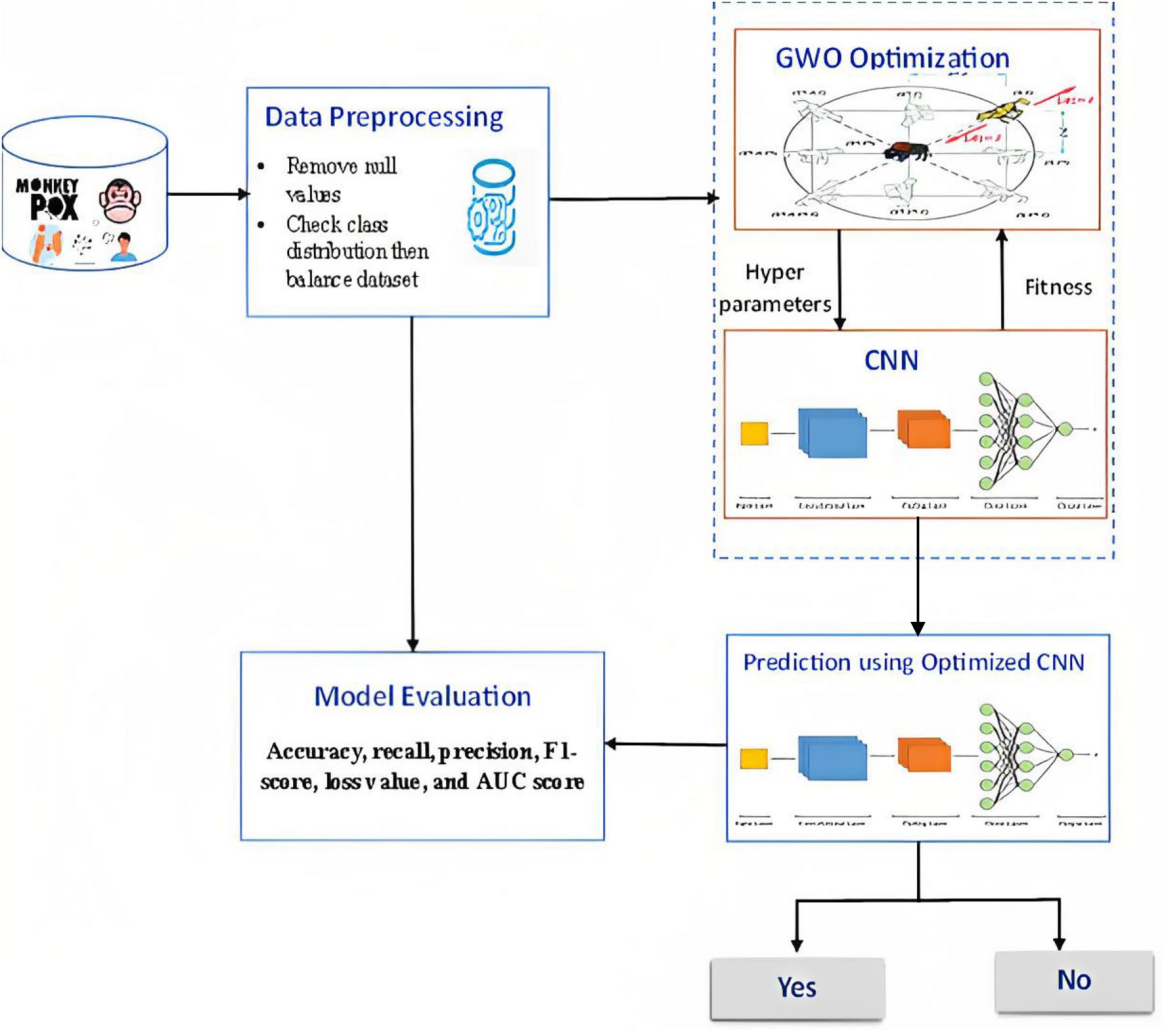
Proposed monkeypox prediction using GWO-based hyperparameter optimized CNN model.

This section presents a discussion on the Monkeypox prediction model, which is composed of four phases: (1) pre-processing of the Monkeypox data, (2) feature selection to identify the most significant symptoms that can enhance the accuracy of Monkeypox diagnosis, (3) Monkeypox prediction using the CNN model, and (4) optimization of the CNN hyperparameters with the GWO algorithm. Figure 2 depicts the four phases of the proposed model.

### Monkeypox dataset description

This section presents an overview of the Monkeypox dataset used in this study. The dataset captures the clinical features of monkeypox infection in humans during the 2022 outbreak in a central London center. The dataset is publicly available on Kaggle at https://www.kaggle.com/datasets/muhammad4hmed/monkeypox-patients-dataset and comprises 25,000 instances with 11 features and a target variable indicating the presence or absence of monkeypox. The features include Fever, Swollen Lymph Nodes, Muscle Aches and Pain, Rectal Pain, Sore Throat, Penile Oedema, Oral Lesions, Solitary Lesion, Swollen Tonsils, HIV Infection, and Sexually Transmitted Infection. The description of each column is as follows^44^:

- Fever: This column represents whether or not the patient has had a fever at some point during their illness. This symptom indicates that the patient body temperature higher than normal.
- Swollen Lymph Nodes: This column represents whether or not the patient has experienced swollen lymph nodes (small, bean-shaped structures in the body that help fight infections) during their illness.
- Muscle Aches and Pain: This column represents whether or not the patient has experienced muscle aches and pain during their illness. These symptoms are often associated with viral infections like monkeypox.
- Rectal Pain: This column may indicate whether the patient is experiencing pain in the rectal area, which could be a symptom of various conditions such as hemorrhoids, anal fissures, or proctitis.
- Sore Throat: This column may indicate whether the patient is experiencing a sore throat, which could be a symptom of various conditions such as tonsillitis, strep throat, or pharyngitis.
- Penile Oedema: This column may indicate whether the patient has swelling in the penis, which could be a symptom of various conditions such as priapism or balanitis.
- Oral Lesions: This column may indicate whether the patient has any lesions or sores in the mouth, which could be a symptom of various conditions such as oral thrush or herpes simplex virus infection.
- Solitary Lesion: This column may indicate whether the patient has a single lesion or sore, which could be a symptom of various conditions such as a cyst, abscess, or skin cancer.
- Swollen Tonsils: This column may indicate whether the patient has enlarged or swollen tonsils, which could be a symptom of various conditions such as tonsillitis or infectious mononucleosis.
- HIV Infection: This column may indicate whether the patient has been diagnosed with human immunodeficiency virus (HIV) infection, which is a viral infection that attacks the immune system.
- Sexually Transmitted Infection: This column may indicate whether the patient has been diagnosed with any sexually transmitted infections (STIs), which are infections spread through sexual contact.
- MonkeyPox: This column may indicate whether the patient has been diagnosed with monkeypox, which is a rare viral disease that can cause skin lesions and other symptoms similar to those of smallpox.

**Figure 3:**
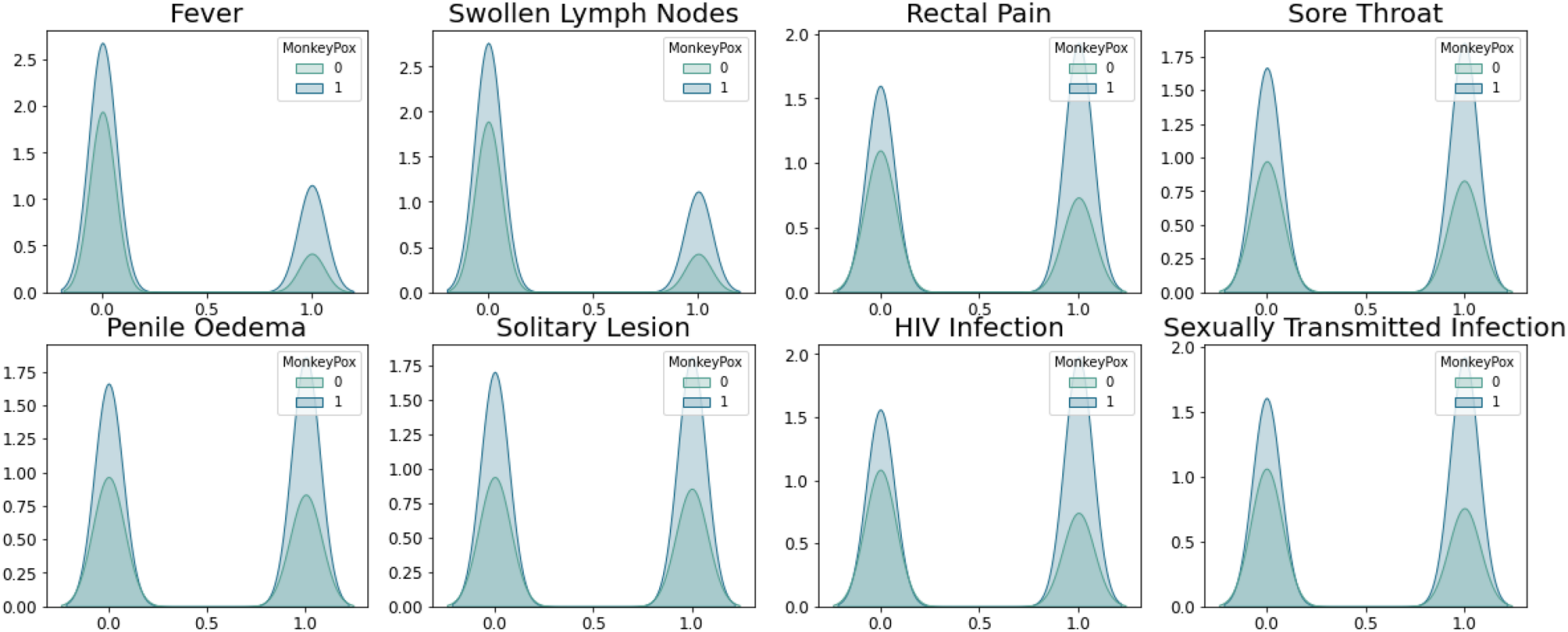
The distribution of the features with each monkeypox class.

The purpose of creating this dataset was to explore the correlations between different factors and the occurrence of monkeypox, as well as to develop a predictive model for diagnosing monkeypox based on these factors. A sample of the Monkeypox dataset is presented in Table 3, and the distribution of features for each Monkeypox class is depicted in Fig. 3.

**Table 3:** A sample of the Monkeypox dataset.

Fever	Swollen lymph nodes	Muscle aches and pain	Rectal pain	Sore throat	Penile oedema	Oral lesions	Solitary lesion	Swollen tonsils	HIV infection	Sexually transmitted infection	MonkeyPox
0	0	0	0	1	1	1	0	1	0	0	0
1	0	0	1	0	1	1	0	0	1	0	1
1	0	0	0	1	1	0	0	0	1	0	1
0	0	0	1	0	0	0	1	1	1	0	1
0	1	0	1	1	1	0	0	1	1	0	1
0	1	0	0	1	0	0	0	0	0	0	0
1	0	0	0	1	0	0	0	0	1	0	1

Figure 3 portrays a clear and distinct separation between the classes of the features, highlighting the pivotal role of features in predicting the target class accurately. This finding strengthens the suitability of the selected features for monkeypox prediction. The observed separation between the feature classes further confirms that the chosen features are essential and informative for the classification of monkeypox. Moreover, the monkeypox dataset exhibits a notable absence of outliers, which is highly desirable in statistical analysis and modeling. Outliers can significantly impact the results and lead to erroneous conclusions, making their absence a crucial advantage in this study. This attribute ensures that the analysis and modeling process is not unduly influenced by extreme values that could skew the results and affect the accuracy of the predictions. However, it is important to note that the dataset is not normally distributed, which can pose potential challenges in certain types of analyses. Non-normality can affect the validity of statistical tests and lead to biased results, making it necessary to address this issue in the data analysis process.

To overcome this challenge, the next subsection of the study outlines the specific steps taken to address non-normality in the dataset and ensure appropriate handling and analysis of the data. These steps include data transformation and the use of appropriate balance datasets technique that are robust to non-normality, ensuring the reliability and accuracy of the study findings.

### The pre-processing of the monkeypox dataset

**Figure 4:**
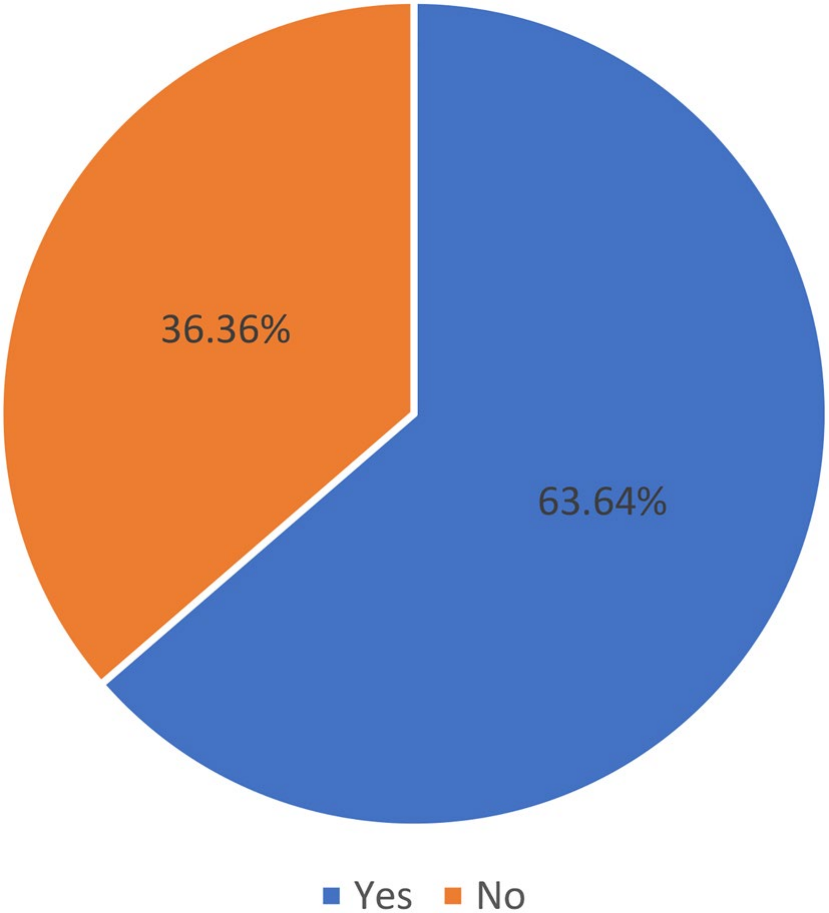
Class distribution.

Cleaning and preprocessing data is crucial in the classification process as it helps to eliminate irrelevant information and noise from the dataset, thereby enhancing the accuracy and efficiency of the classification model. In the case of the Monkeypox dataset, missing values are removed during the data cleaning process. However, the dataset is not properly distributed as illustrated in Fig. 4. To balance the data, one of the widely used techniques is SMOTEEN^45^. This technique combines SMOTE (Synthetic Minority Over-sampling Technique) and ENN (Edited Nearest Neighbors) to create a balanced dataset that is less prone to noise.

### Feature selection

**Figure 5:**
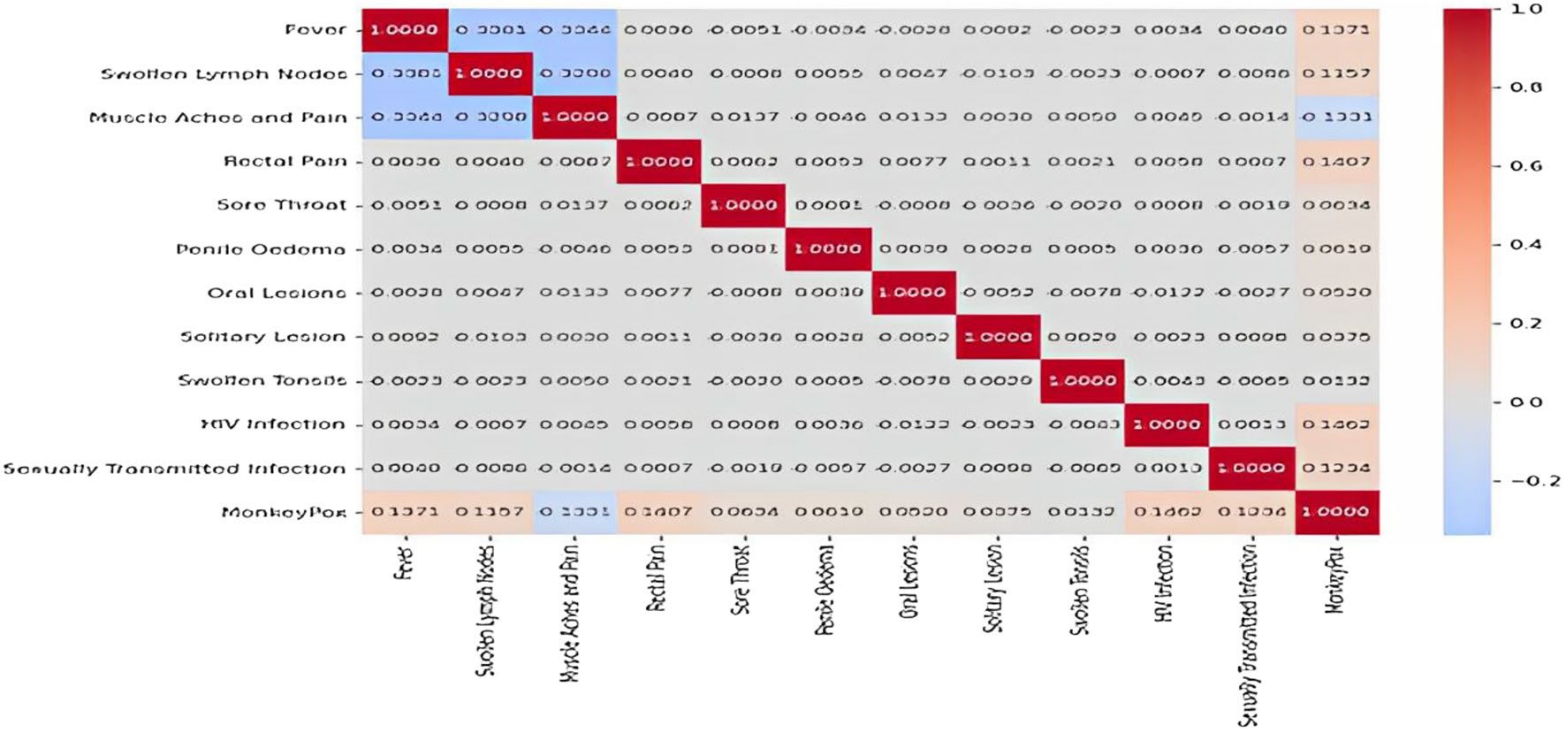
The heatmap of the Monkeypox dataset.

After preprocessing the monkeypox dataset by removing null values and balancing the data using the SMOTEEN algorithm, the selection of most important features is a critical step in developing prediction models as it directly affects the performance of the models^46^. Correlation analysis is an effective method to identify the dependence among the features of a dataset. By identifying which variables are strongly correlated with the target variable, it is possible to select the most important features and reduce the number of variables included in the model. This, in turn, can improve the model's accuracy and reduce overfitting. A correlation matrix is a tool used to visualize the correlation coefficients between pairs of variables in a dataset. The heatmap in Fig. 5 shows the correlation matrix for the monkeypox dataset. Correlation coefficients range from − 1 to + 1 and indicate the strength and direction of the relationship between two variables. A value of + 1 indicates a perfect positive correlation, -1 indicates a perfect negative correlation, and 0 indicates no correlation between the variables.

### Monkeypox prediction using CNN

**Figure 6:**
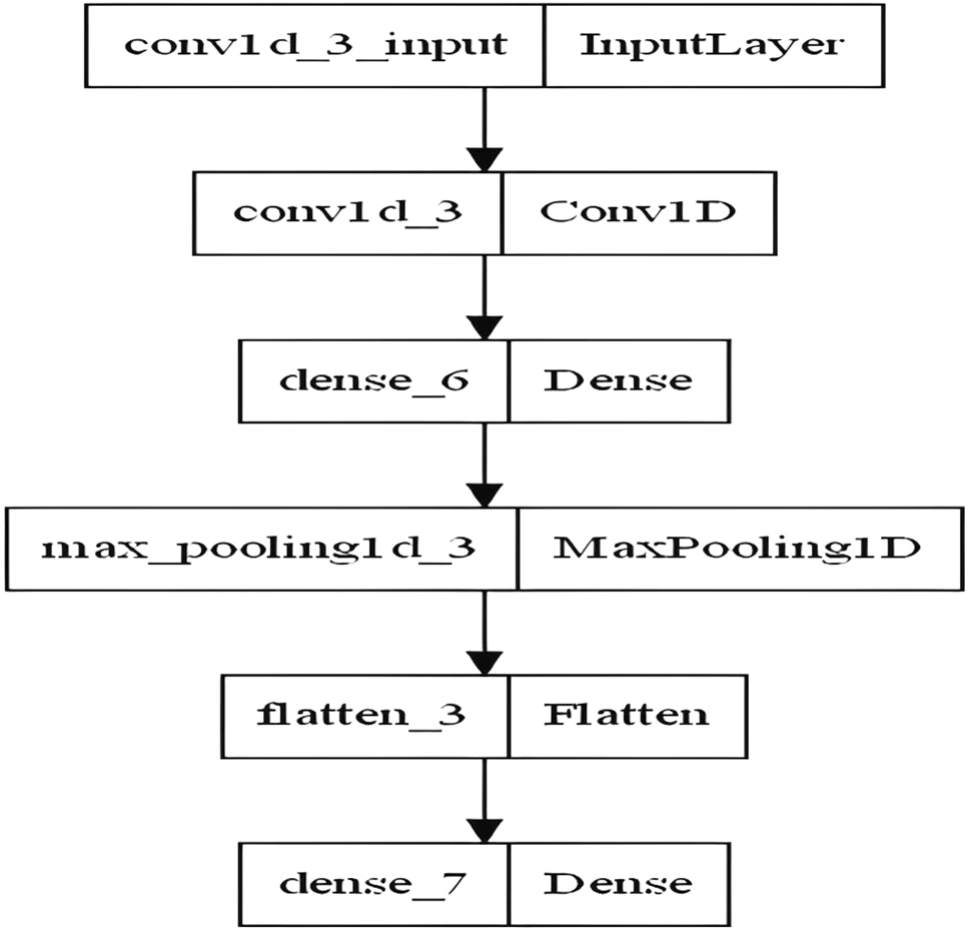
The proposed CNN architecture.

Inspired by the interesting features of deep networks, in this phase, the CNN model is utilized for monkeypox prediction. after preprocessing the monkeypox dataset and detecting the most frequent features. The CNN architecture is shown in Fig. 6, which consists of an input layer that is A 1D convolutional layer with kernel size of 2, and ReLU activation function, a hidden layer that is a dense layer with ReLU activation function, max pooling layer that is a pooling layer with default pool size of 2, flatten layer that is a layer to flatten the output from the previous layer, and output layer that is a dense layer with 1 neuron and sigmoid activation function.

In this study, alternative Convolutional Neural Network (CNN) architectures were evaluated. The selection of the specific CNN layers employed in the defined architecture was based on the characteristics of the dataset utilized in the experiment. The distinct CNN architectures were optimized through the utilization of performance metrics, including accuracy, loss, and validation results.

To ensure that the system is not underfitted or overfitted, we employed various techniques during the training and evaluation stages of the model. To prevent underfitting, we ensured that the model was complex enough to capture the underlying patterns in the data. This was achieved by selecting an appropriate model architecture and hyperparameters. We also augmented the dataset with additional samples and performed data preprocessing to ensure that the data was representative of the problem domain.

To prevent overfitting, we used techniques such as early stopping and regularization during the training stage. Early stopping was employed to stop the training process once the validation error no longer improves, thereby preventing the model from memorizing the training data. Regularization was also used to reduce the complexity of the model and prevent it from overfitting to the training data. Additionally, we evaluated the performance of the model on a separate test set to ensure that it generalized well to unseen data. If the model performed well on the test set, it was an indication that it was not overfitted to the training data. A balance between model complexity and generalization performance was sought to ensure that the system was not underfitted or overfitted.

### Hyperparameters optimization of CNN using the GWO algorithm

**Figure 7:**
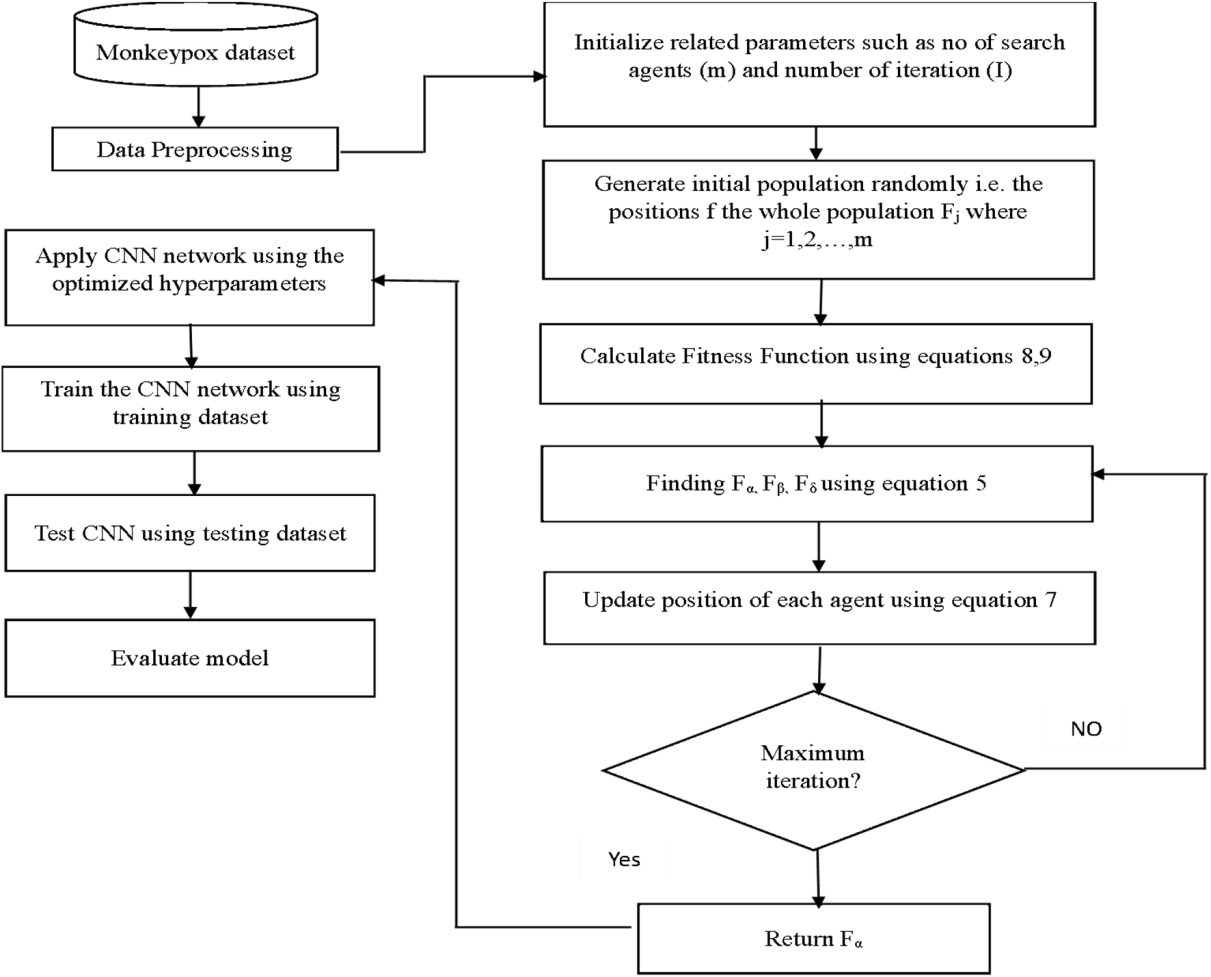
The flowchart of the proposed monkeypox prediction model that utilizes the GWO optimization technique to fine-tune the hyperparameters of the CNN.


After determining the CNN model that we will use in our design, the scores were observed by changing the hyperparameters of the selected model. Especially optimal parameters have a direct impact on the accuracy of monkeypox detection as the deep learning architecture is represented by parameterized functions. To find the optimal values for various hyperparameters such as learning rate, batch size, number of layers, and filter size, the GWO algorithm has been applied to various optimization problems, including parameter tuning for CNNs. The GWO optimization algorithm can be utilized for CNN parameter tuning by defining the search space for each hyperparameter and searching for the optimal set of hyperparameters. In this approach, the hyperparameters are considered decision variables, and the objective function is the classification accuracy, which is the performance metric of CNN^47^. The GWO algorithm commences by initializing a population of grey wolves, each of which represents a potential solution^48^. The CNN is trained on a training dataset and its performance is evaluated on a validation dataset to determine the efficacy of each solution. The position of each grey wolf is then updated based on the performance of each solution using a set of formulas that simulate the social behavior of grey wolves in nature. This iterative process continues until a stopping criterion is met, such as a maximum number of iterations or a minimal improvement in the performance metric. The best solution identified by the GWO algorithm corresponds to the optimal set of hyperparameters for the CNN. Algorithm 1 represents the pseudocode of the proposed monkeypox prediction using a GWO-based hyperparameter-optimized CNN algorithm. Figure 7 represents the flowchart of the proposed monkeypox prediction using a GWO-based hyperparameter-optimized CNN algorithm.

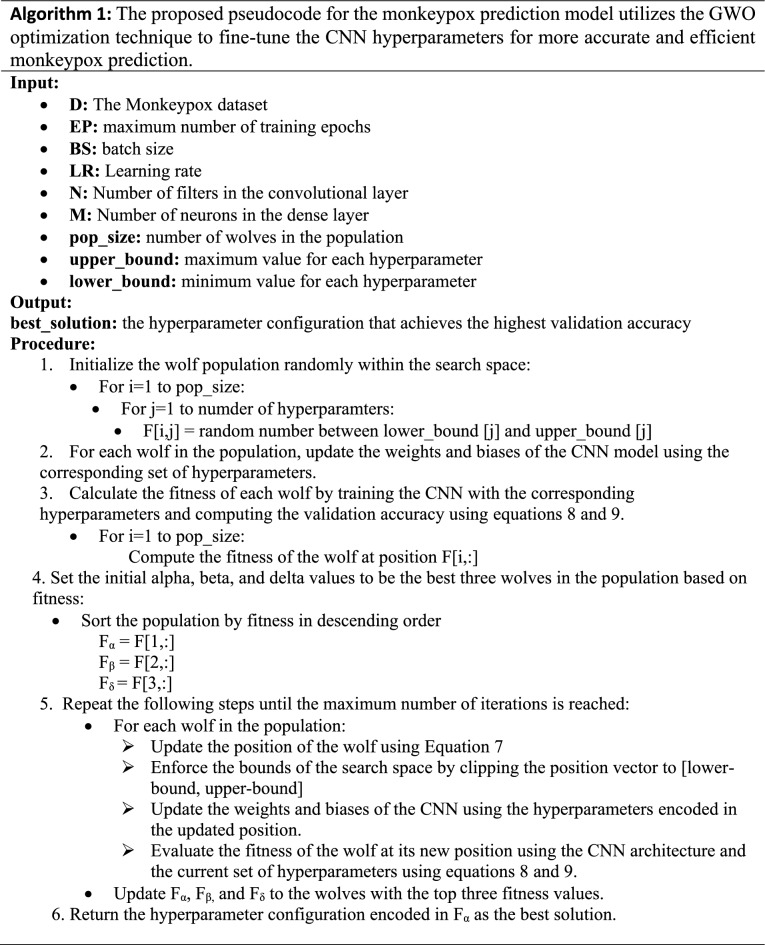


### Monkeypox model evaluation

Following the completion of the training process of our model, the model must be verified and tested. The performance Monkeypox prediction model is validated using known performance metrics such as accuracy, recall, precision, F1-score^49^, the Categorical cross entropy loss (loss) value, and the Area Under the Curve (AUC) score^50,51^ as in Eqs. (6, 10, 11, 12, 13 and 14) respectively.

10 $$\mathrm{Accuracy}=\frac{\mathrm{TP }+\mathrm{ TN}}{\mathrm{TP }+\mathrm{ FP }+\mathrm{ TN }+\mathrm{ FN}},$$

11 $$\mathrm{Recall}=\frac{\mathrm{TP }}{\mathrm{TP }+\mathrm{ FN}},$$

12 $$\mathrm{Precision}=\frac{\mathrm{TP }}{\mathrm{TP }+\mathrm{ FP}},$$

13 $$\mathrm{F}1 -\mathrm{ score }=2* \frac{\left(\mathrm{Precision }\times \mathrm{ Recall}\right)}{\left(\mathrm{Precision }+\mathrm{ Recall}\right)},$$

14 $$\mathrm{Loss}={\sum }_{k=0}^{n}{\mathrm{z}}_{k.}\mathrm{log}{\widehat{z}}_{k}loss=-{\sum }_{k=0}^{n}{\mathrm{z}}_{k.}\mathrm{log}{\widehat{z}}_{k,}$$

where, TP, TN, FN, and FP are truly positive, true Negative, False Negative, and False Positive numbers respectively. n is the number of classes, ${\widehat{z}}_{k}$ is the model predicted value for kth class, ${\mathrm{z}}_{k.}$ is the corresponding target value.

Area Under the Curve (AUC) is a crucial metric used in classification tasks, representing the area under the Receiver Operating Characteristic (ROC) curve. A value close to 1.00 implies good classification performance, while a score greater than 0.50 is considered acceptable for the model.

### Ethical statement

This article does not contain any studies with human participants or animals performed by any of the authors.

## Experimental results and analysis

In this section, we have conducted experiments to assess the performance of the monkeypox prediction model. As mentioned before, to construct the prediction model the monkeypox patient’s dataset that describes the clinical features of monkeypox infection in humans in London. We conducted our experiments on with 3 GHz AMD Ryzen 7 computer with 16 GB main memory and a 64-bit Windows 10 operating system. The experiment is carried out using the Python programming language.

The effectiveness of a deep learning model is heavily reliant on the quality of data and the methodology employed in utilizing the data^52^. Consequently, evaluating the impact of data preprocessing on the performance of machine learning models is crucial. To enhance the classifier's performance, we began by eliminating the missing values from the Monkeypox dataset. We then assessed the distribution of the entire dataset to verify the class distribution. Following that, we investigated how the selection of the most critical features influenced classification performance. Finally, we utilized the GWO algorithm to optimize the CNN hyperparameters.

The proposed method for monkeypox prediction was analyzed to obtain a conclusive assessment of the trained model. This evaluation process was performed both before and after the application of data preprocessing, feature selection, and hyperparameter optimization using GWO.

### Experiment I

**Figure 8:**
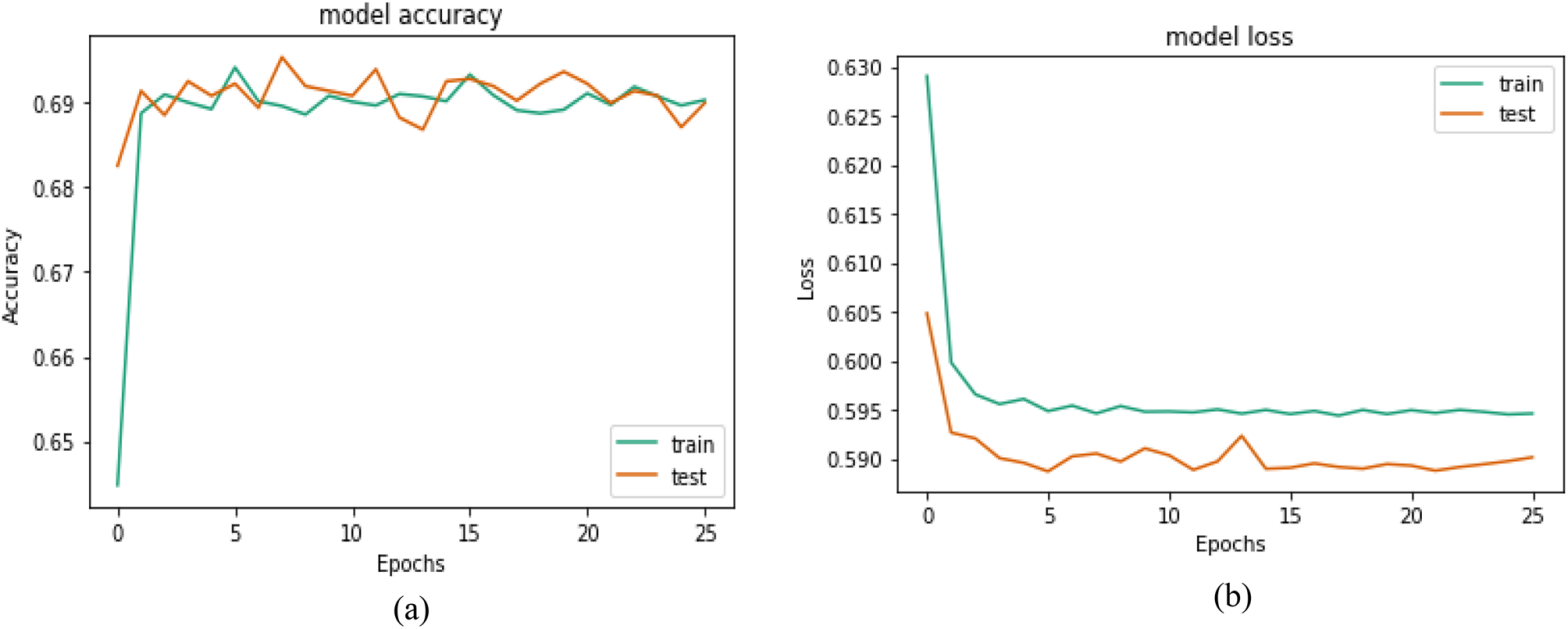
Accuracy and loss comparisons for the testing and training datasets of the monkeypox patients.

The CNN is run in the first experiment without data preprocessing, feature selection, and hyperparameter optimization using GWO. Table 4 shows the training performance of the model. The testing results are 68.826%, 70.569%, 87.880%, 78.279%, and 0. 61.475% for Accuracy, Precision, Recall, F1 Score, and AUC Score, respectively. Accuracy and loss comparisons for the testing and training datasets of monkeypox patients are shown in Fig. 8.

**Table 4:** The performance results for the CNN model on the Monkeypox dataset.

Model	Evaluation metrics				
	Accuracy	Precision	Recall	F1 score	AUC score
CNN	68.826	70.569	87.880	78.279	61.475

**Figure 9:**
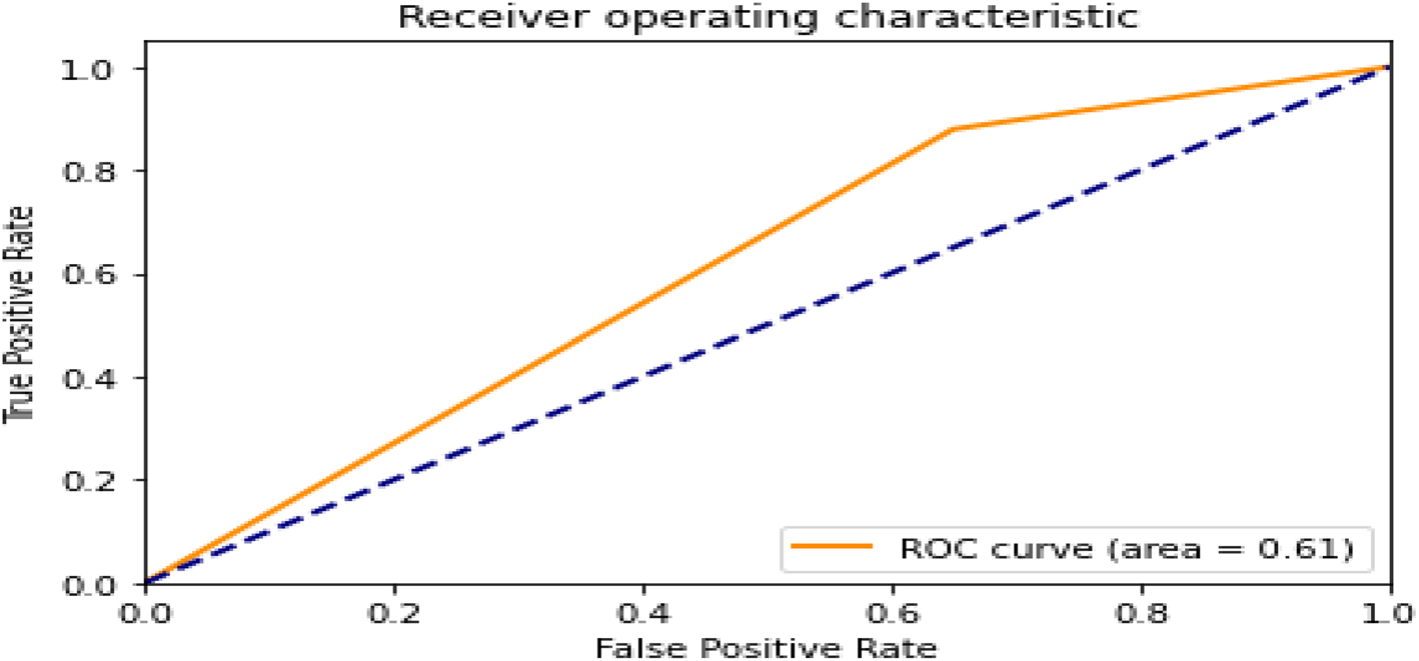
The ROC curve of the CNN model.

The ROC curve obtained after the prediction with the CNN model on the test dataset is shown in Fig. 9. It can be seen from the ROC curve that the AUC score of 0.61 suggests that the model is performing better than random guessing, but its performance may not be particularly strong.

### Experiment II

In the second experiment, the CNN was used after preprocessing the monkeypox dataset by removing null values and balancing the data using the SMOTEEN algorithm, then identifying the top most frequent features in the dataset and using the GWO algorithm for CNN hyperparameter optimization.

**Figure 10:**
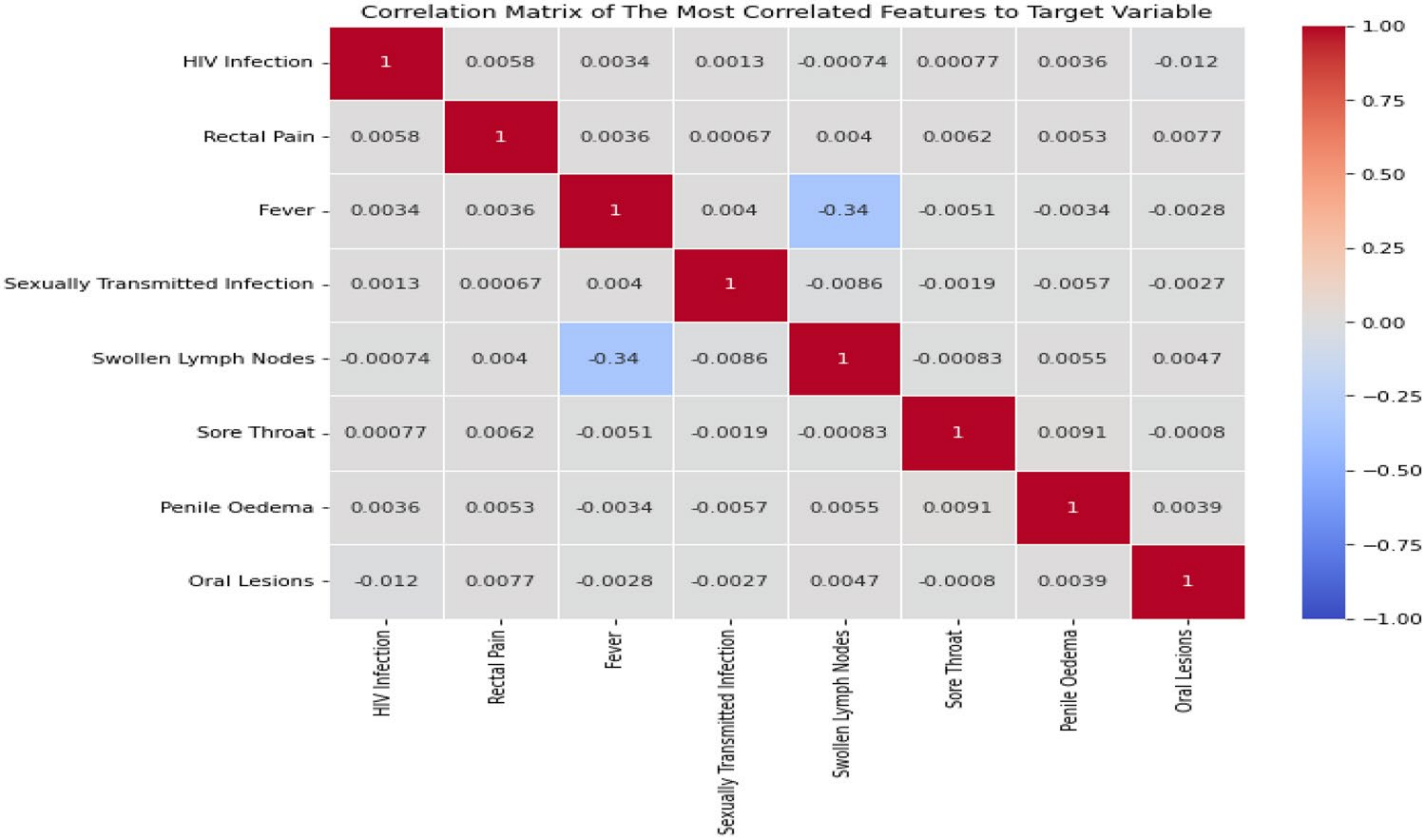
Correlation matrix of the most correlated features that affected Monkeypox diagnoses decision.

**Figure 11:**
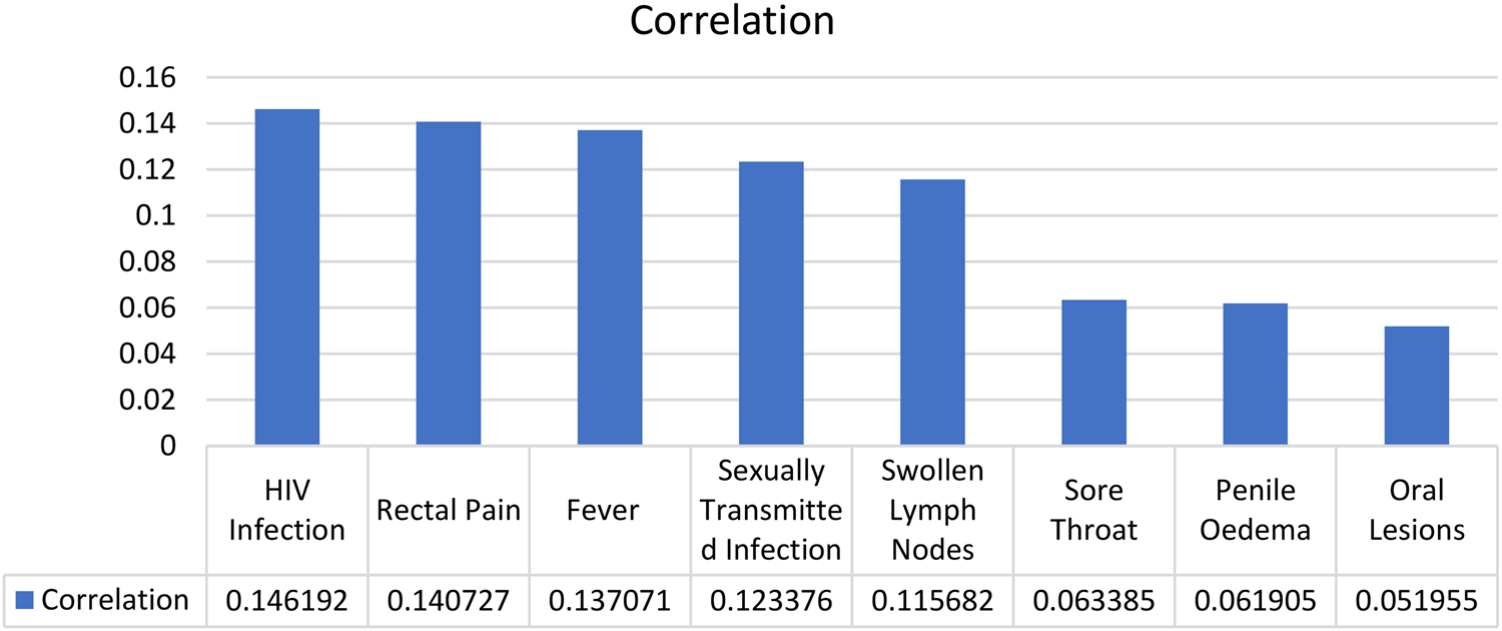
Correlation of the most correlated features to the target variable.

To identify the most significant features that affected the Monkeypox diagnoses decision. The proposed model detected the top most frequent features in the dataset, which are HIV Infection Rectal Pain, Fever, Sexually Transmitted Infection, Swollen Lymph Nodes, Sore Throat, Penile Oedema, and Oral Lesions as shown in Figs. 10 and 11.

After preprocessing the monkeypox dataset and detecting the most frequent features, the CNN model is utilized for monkeypox prediction. However, the choice of CNN architecture and hyperparameter settings significantly affects accuracy and convergence speed. Manual tuning of these hyperparameters is a time-consuming and computationally expensive task. Therefore, an automated approach is required to produce the best CNN architecture with minimal human intervention. In this study, we utilized the GWO algorithm to optimize the CNN hyperparameters within a predefined search space. The hyperparameters associated with the CNN and their ranges used for experiments are shown in Table 5. To investigate the effects of the optimization performance, we varied the number of search agents (population size) and a maximum number of iterations.

**Table 5:** Domains of hyperparameters.

Learning rate	Number of epochs	Batch size	Number of filters in the convolutional layer	Number of neurons in the dense layer
[0.001, 1.0]	[50, 200]	[32, 128]	[64, 256]	[32, 128]

Table 5 presents the results of using the GWO to optimize the hyperparameters of a CNN for a Monkeypox prediction. Three runs of the algorithm were performed with different GWO combinations of population size and number of iterations and their corresponding selected CNN parameters and fitness scores.

Table 6 shows the selected hyperparameters of a Convolutional Neural Network (CNN) using three different runs of the Grey Wolf Optimizer (GWO) algorithm. The table consists of four columns: GWO parameters, Selected CNN parameters, and Fitness Score. In the GWO parameters column, each run of the GWO optimizer used different parameters such as population size, learning rate (LR), exploration probability (EP), batch size (BS), number of hidden layers (N), and number of neurons in each layer (M). In the Selected CNN parameters column, the hyperparameters chosen by GWO for the CNN model are listed. These include LR, EP, BS, N, and M, which represent the learning rate, exploration probability, batch size, number of hidden layers, and number of neurons in each layer, respectively. Finally, the Fitness Score column shows the performance of the CNN model in terms of its accuracy. The fitness score is a measure of how well the CNN was able to classify the input data, with higher scores indicating better performance.

**Table 6:** The selected hyperparameters of a CNN using three different runs of GWO optimizer.

GWO parameters		Selected CNN parameters					Fitness score
		LR	EP	BS	N	M	
Run1	Population size = 50	0.001	60	64	85	40	0.94632
	No of iterations = 10						
Run2	Population size = 60	0.08	60	64	168	32	0.91252
	No of iterations = 20						
Run3	Population size = 70	0.04	80	50	85	32	0.91948
	No of iterations = 30						

Where *LR* Learning Rate, *EP* Epochs, *BS* Batch Size, *N* Number of filters in the convolutional layer, *M* Number of neurons in the dense layer.

Based on the results in Table 4, it can be observed that the highest fitness value of 0.94632 was obtained was achieved with a population size of 50, learning rate of 0.001, 60 epochs, batch size of 64, 85 filters in the conventional layer, and 40 neurons in the dense layer with 10 iterations. As we can see from the table, each run of the GWO optimizer resulted in different sets of hyperparameters being selected for the CNN model, which in turn resulted in different fitness scores. This indicates that the performance of the CNN is highly dependent on the hyperparameters selected, and that tuning these hyperparameters using an optimizer such as GWO can lead to improved accuracy.

The evaluation metrics for various CNN models with different hyperparameters optimized by the GWO algorithm are presented in Table 7. These hyperparameters were optimized using different combinations of population size and number of iterations. The performance of the models was evaluated based on several metrics, including accuracy, precision, recall, F1 score, and AUC score, as described in Sect. 5.6.

**Table 7:** The performance of the CNN model optimized using the GWO algorithm with different combinations of population size and number of iterations.

GWO with different combinations of parameters	Evaluation metrics for CNN model				
	Accuracy	Precision	Recall	F1 Score	AUC Score
Run 1	95.312	95.638	98.145	96.875	92.686
Run 2	93.465	93.806	97.722	95.724	89.256
Run 3	93.276	93.425	97.914	95.617	88.673

From the results in Table 7, it can be noticed that The CNN model trained with parameters resulting from GWO with a population size of 50 and 10 iterations achieved the highest accuracy (95.312%). This model also has the highest precision (95.638%) and recall (98.145%) values, indicating that it performed well in correctly identifying positive cases and minimizing false positives. Additionally, the model achieved a high F1 score of 96.875% and an AUC score of 92.686%. The study findings suggest that the hyperparameters selected by GWO, namely population size, learning rate, and number of iterations, can considerably influence the CNN's performance. Furthermore, it is noteworthy that the population size and number of iterations used in the optimization process can also affect the final outcomes, as seen by the varying fitness scores achieved in each run. Interestingly, the research also reveals that increasing the population size and number of iterations did not necessarily result in improved fitness values.

**Figure 12:**
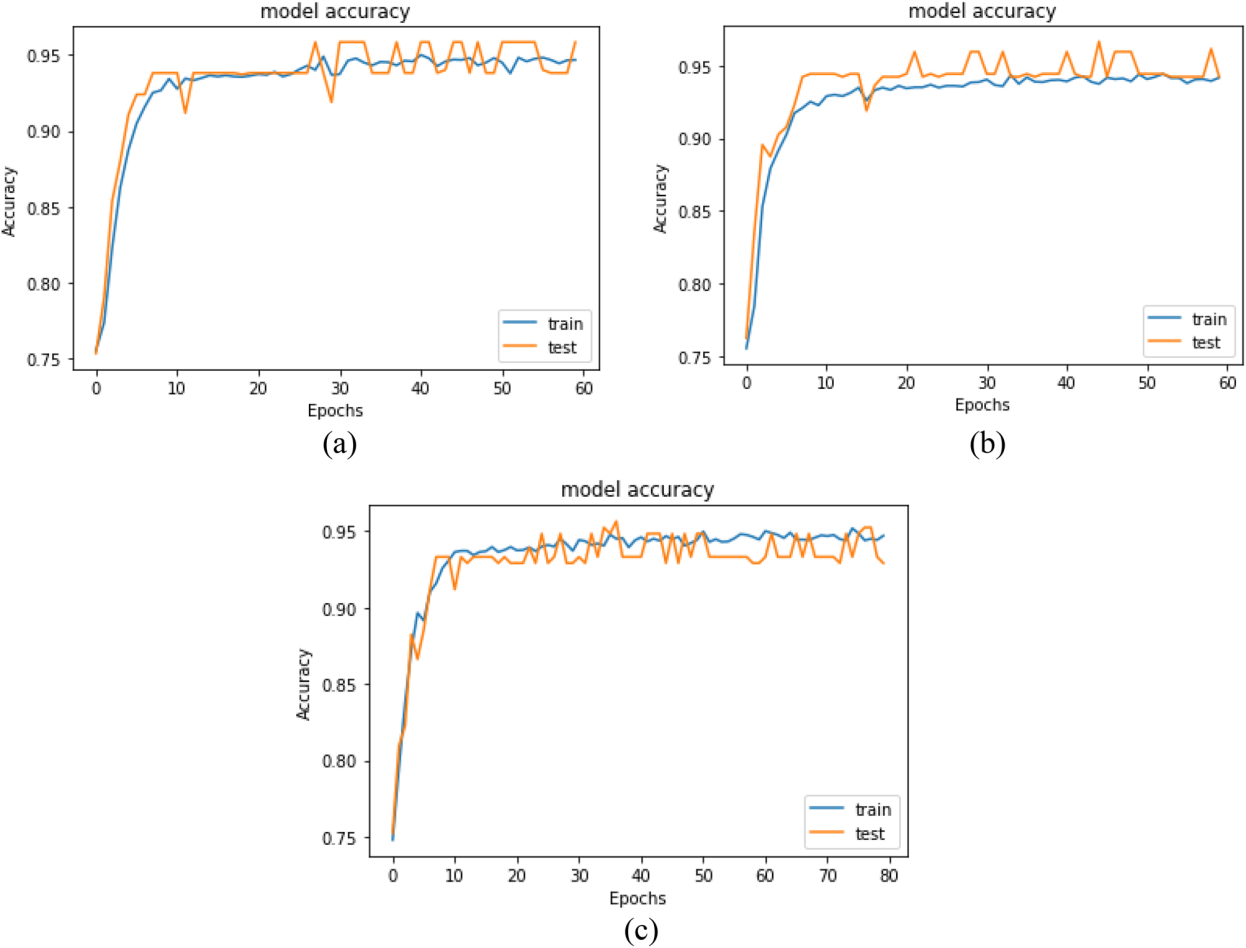
The CNN accuracy comparison for the testing and training datasets of the monkeypox patients after applying GWO. (**a**) The CNN model trained with parameters resulting from GWO with a population size of 50 and 10 iterations. (**b**) The CNN model trained with parameters resulting from GWO with a population size of 60 and 20 iterations. (**c**) The CNN model trained with parameters resulting from GWO with a population size of 70 and 30 iterations.

**Figure 13:**
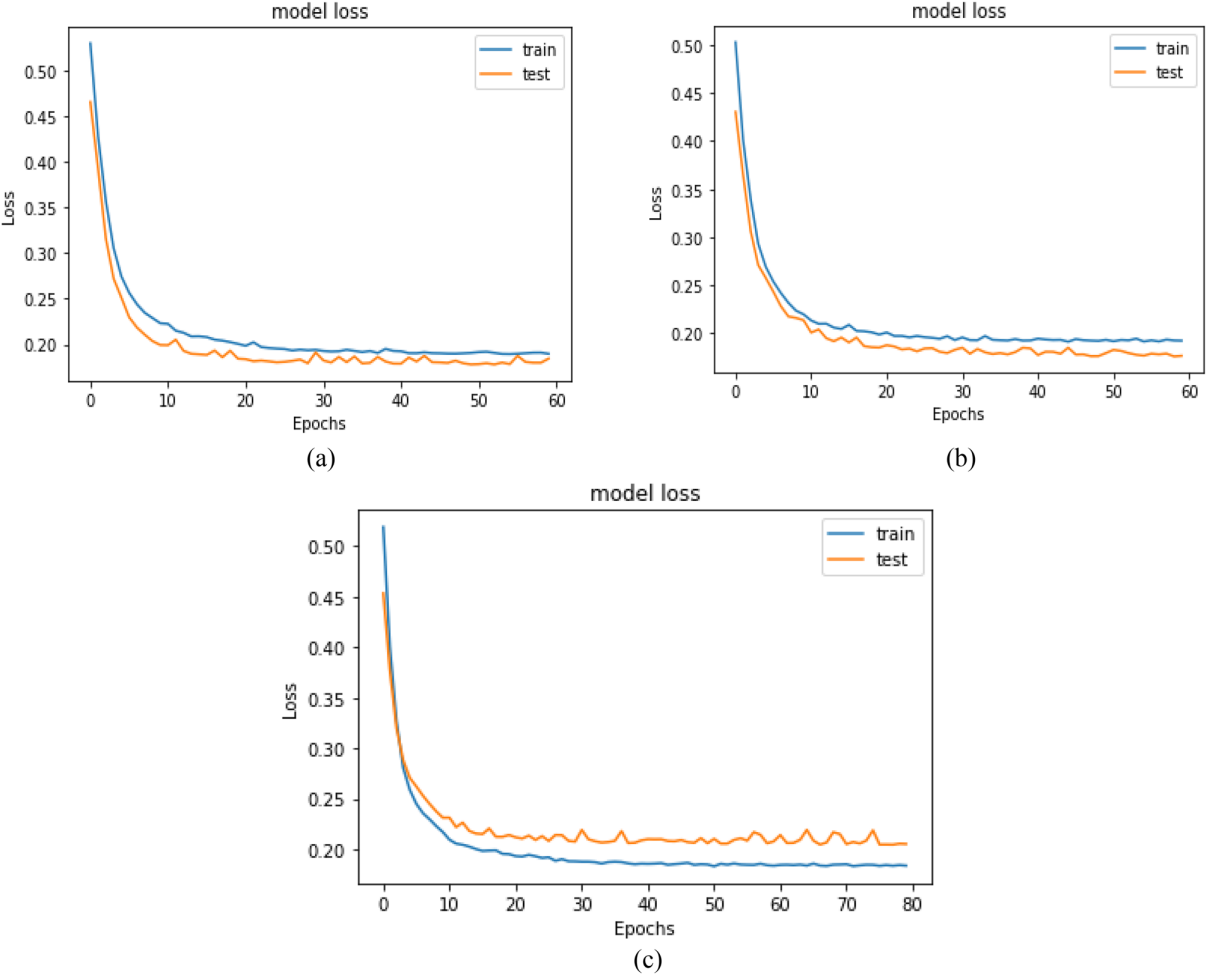
The CNN loss comparison for the testing and training datasets of the monkeypox patients after applying GWO. (**a**) The CNN model trained with parameters resulting from GWO with a population size of 50 and 10 iterations. (**b**) The CNN model trained with parameters resulting from GWO with a population size of 60 and 20 iterations. (**c**) The CNN model trained with parameters resulting from GWO with a population size of 70 and 30 iterations.

A comparison between the CNN model accuracy and loss for the testing and training datasets of monkeypox patients is shown in Figs. 12 and 13. The CNN model was applied with different hyperparameters that were optimized using the GWO algorithm with different combinations of population size and number of iterations.

**Figure 14:**
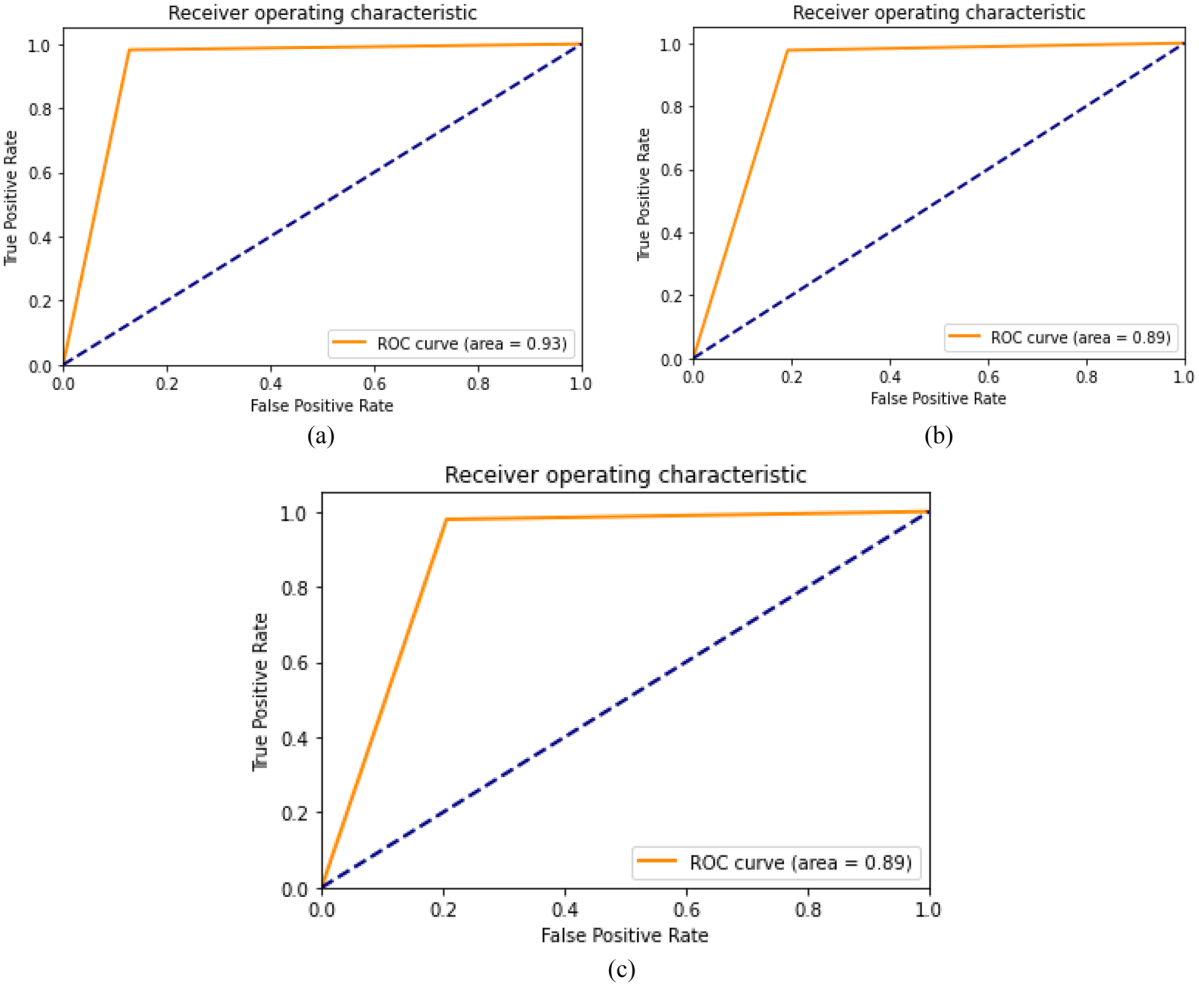
CNN model ROC curve after applying GWO. (**a**) The CNN model trained with parameters resulting from GWO with a population size of 50 and 10 iterations. (**b**) The CNN model trained with parameters resulting from GWO with a population size of 60 and 20 iterations. (**c**) The CNN model trained with parameters resulting from GWO with a population size of 70 and 30 iterations.

Figure 14 shows the ROC curve generated by the CNN model using parameters optimized with the GWO algorithm on the test dataset. The GWO algorithm was used with various combinations of population size and number of iterations. The ROC curve with an AUC score of 0.93, corresponds to the CNN model trained using the GWO optimizer with a population size of 50 and 10 iterations. This result suggests that the model possesses high discriminatory capability and can effectively differentiate between positive and negative classes. Additionally, the curve's proximity to the top-left corner of the plot signifies a high true positive rate and a low false positive rate. It can be seen from the ROC curve that the AUC score of 0.61 suggests that the model is performing better than random guessing, but its performance may not be particularly strong.

**Figure 15:**
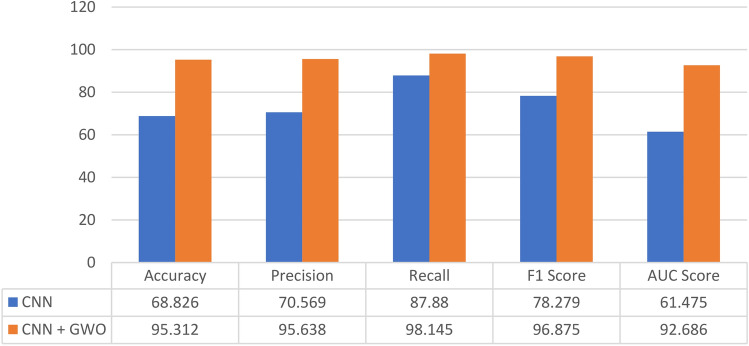
The performance comparison for two models: a CNN model and a CNN model trained with parameters resulting from GWO optimizer with a population size of 50 and 10 iterations.

Figure 15 shows the evaluation metrics for two models: a CNN model and a CNN model trained with parameters resulting from a GWO optimizer with a population size of 50 and 10 iterations. The CNN model was trained with a learning rate of 0.001, 60 epochs, batch size of 64, 85 filters in the conventional layer, and 40 neurons in the dense layer.

The study results indicate that the CNN model's performance is lower than the CNN model with GWO optimizer. The CNN model has an accuracy of 68.826%, while the CNN model with GWO has an accuracy of 95.312%. The precision, recall, F1 score, and AUC score of the CNN model with GWO are also significantly higher than the CNN model. The findings demonstrate that incorporating the GWO optimizer to the CNN model has substantially improved its performance in terms of all evaluation metrics. The accuracy, precision, recall, F1 score, and AUC score have all increased when utilizing the GWO-optimized model compared to the non-optimized model. Specifically, the AUC score has improved from 61.475 to 92.686, indicating a significant improvement in the model's ability to distinguish between positive and negative classes. The enhancement in other metrics such as recall, precision, and F1 score suggests that the GWO optimizer has resulted in better performance in accurately identifying and classifying positive instances. Overall, the findings demonstrate that GWO optimization can be a valuable approach to improving the performance of CNN models for monkeypox classification tasks.

## Discussion

While the proposed approach of utilizing CNNs to classify monkeypox skin lesions has shown promise, several challenges need to be addressed:

1. Data availability: The availability of large and diverse datasets of monkeypox skin lesions is limited, which can affect the CNN model's performance.
2. Data quality: The quality of the available monkeypox skin lesion may vary, which can affect the CNN model's accuracy.
3. Dataset bias: The dataset used to train the CNN model may not be representative of the overall population, which can lead to biased results.
4. Overfitting: Overfitting can occur when the CNN model learns the training data too well, resulting in poor generalization to new and unseen data.
5. Interpretability: CNN models are often considered to be black boxes, which can make it challenging to interpret the model's decisions.
6. Transfer learning: The effectiveness of transfer learning, which is used to fine-tune the pre-trained CNN models, can vary depending on the similarity between the source and target datasets.
7. Optimization: The optimization of the CNN model, such as selecting the best hyperparameters and optimization algorithm, can be time-consuming and require significant computational resources.
8. Error analysis: The identification and analysis of errors made by the CNN model can be challenging, making it difficult to identify areas for improvement.
9. Integration with healthcare systems: The integration of the proposed approach with existing healthcare systems and workflows may require significant changes and investments.
10. Cost: The cost of implementing the proposed approach, including the necessary technology and infrastructure, may be prohibitive in some settings.
11. Performance in real-world settings: The performance of the proposed approach in real-world settings may differ from the results obtained in the study due to various factors such as patient variability and environmental conditions.
12. Confounding factors: Other factors, such as underlying medical conditions or medication usage, may affect the appearance of monkeypox skin lesions, which can impact the CNN model's accuracy.
13. Limited generalizability: The proposed approach's generalizability to other skin diseases or medical conditions that require visual inspection and diagnosis may be limited.

It is possible that the CNN-based approach used in the monkeypox study could be adapted to other types of data, such as clinical and images data, to improve disease diagnosis and surveillance. For example, the approach could be used to analyze patterns in lung function tests or blood biomarkers to help diagnose and predict the progression of lung diseases^53^ such as COPD or pulmonary fibrosis, as discussed in the article on screening lung diseases. Similarly, the approach could be applied to clinical and images data from chest X-ray images to improve the accuracy of machine learning-based diagnosis of COVID-19^54^, as discussed in the article on a machine learning-based framework for COVID-19 diagnosis. However, further research would be needed to determine the feasibility and effectiveness of such applications.

## Future direction

The potential for utilizing CNNs to classify monkeypox skin lesions is vast, and several avenues for future work can be explored. Some of these include:

- Improved data collection and annotation: In this study, we utilized a small clinical dataset with limited annotations. Future work can involve larger datasets with better annotations, which can help in improving the accuracy of the model.
- Transfer learning: Transfer learning is a powerful technique that allows the use of pre-trained models for new tasks with minimal training data. Future work can involve the use of transfer learning to improve the accuracy of the model.
- Multi-class classification: In this study, we focused on binary classification (positive or negative for monkeypox). Future work can involve multi-class classification to classify different types of skin lesions and diseases.
- Integration with clinical decision-making: The integration of AI models with clinical decision-making can have significant benefits for patient care. Future work can involve the integration of the monkeypox skin lesion classification model with clinical decision-making tools to aid in diagnosis and treatment.
- Generalization of other skin diseases: The use of CNNs can be extended to classify other skin diseases. Future work can involve the development of models for other skin diseases, such as chickenpox, herpes, and shingles.
- Integration with telemedicine: The use of AI models can be integrated with telemedicine platforms to improve access to healthcare, especially in areas with limited access to dermatologists. Future work can involve the development of telemedicine platforms that can integrate with the monkeypox skin lesion classification model.
- Explainability and interpretability: AI models can sometimes be black boxes, making it challenging to understand the rationale behind the decisions they make. Future work can involve the development of explainable AI models that can provide insights into the decision-making process of the model.

## Conclusion

Monkeypox is a viral disease characterized by skin lesions and rashes, often challenging to diagnose accurately through visual inspection. This study proposes the use of CNNs to classify monkeypox skin lesions. The approach was evaluated on a test set using accuracy, precision, recall, F1-score, and AUC score achieving 95.3% accuracy, surpassing other methods. Furthermore, the CNN model was optimized using the GWO algorithm, resulting in a significant improvement in accuracy, precision, recall, F1-score, and AUC score compared to the non-optimized model. The GWO optimization can enhance the performance of CNN models on similar tasks. This approach has the potential to improve monkeypox diagnosis and surveillance, particularly in resource-limited settings, with crucial public health implications. In summary, the study highlights that CNNs and GWO optimization can significantly improve the accuracy of monkeypox skin lesion classification and enhance monkeypox diagnosis and control.

## Data Availability

The data that support the findings of this study are available at https://www.kaggle.com/datasets/muhammad4hmed/monkeypox-patients-dataset.
